# A New Dermal Substitute Containing Polyvinyl Alcohol with Silver Nanoparticles and Collagen with Hyaluronic Acid: In Vitro and In Vivo Approaches

**DOI:** 10.3390/antibiotics10060742

**Published:** 2021-06-19

**Authors:** Dario Mendes Júnior, Moema A. Hausen, Jéssica Asami, Akemi M. Higa, Fabio L. Leite, Giovanni P. Mambrini, Andre L. Rossi, Daniel Komatsu, Eliana A. de Rezende Duek

**Affiliations:** 1 Faculty of Medical Sciences and Health, Pontifical Catholic University of São Paulo (PUC/SP), São Paulo 18030-070, Brazil; mendeda3@gmail.com (D.M.J.); mahausen@pucsp.br (M.A.H.); dkomatsu@pucsp.br (D.K.); 2 Faculty of Mechanical Engineering, State University of Campinas (UNICAMP), São Paulo 13083-860, Brazil; jessicaasami@gmail.com; 3 Instituto de Medicina Tropical, Universidade de São Paulo (USP), São Paulo 05403-000, Brazil; akemi.higa@usp.br; 4 Department of Physics, Chemistry and Mathematics, Federal University of São Carlos (UFSCar), São Paulo 18052-780, Brazil; fabioleite@ufscar.br (F.L.L.); gpmambrini@ufscar.br (G.P.M.); 5 Applied Physics Department, Brazilian Center of Physics Research (CBPF), Rio de Janeiro 22290-180, Brazil; andrelinharesrossi@gmail.com

**Keywords:** polyvinyl alcohol, silver nanoparticles, dermal substitute, wound healing

## Abstract

The experimental use of poly (alcohol-vinyl) (PVA) as a skin curative is increasing widely. However, the use of this hydrogel is challenging due to its favorable properties for microbiota growth. The association with silver nanoparticles (AgNPs) as an antimicrobial agent turns the match for PVA as a dressing, as it focuses on creating a physical barrier to avoid wound dehydration. When associated with extracellular components, such as the collagen matrix, the device obtained can create the desired biological conditions to act as a skin substitute. This study aimed to analyze the anti-microbiological activity and the in vitro and in vivo responses of a bilaminar device of PVA containing AgNPs associated with a membrane of collagen–hyaluronic acid (col-HA). Additionally, mesenchymal stem cells were cultured in the device to evaluate in vitro responses and in vivo immunomodulatory and healing behavior. The device morphology revealed a porous pattern that favored water retention and in vitro cell adhesion. Controlled wounds in the dorsal back of rat skins revealed a striking skin remodeling with new epidermis fulfilling all previously injured areas after 14 and 28 days. No infections or significant inflammations were observed, despite increased angiogenesis, and no fibrosis-markers were identified as compared to controls. Although few antibacterial activities were obtained, the addition of AgNPs prevented fungal growth. All results demonstrated that the combination of the components used here as a dermal device, chosen according to previous miscellany studies of low/mid-cost biomaterials, can promote skin protection avoiding infections and dehydration, minimize the typical wound inflammatory responses, and favor the cellular healing responses, features that give rise to further clinical trials of the device here developed

## 1. Introduction

Polyvinyl alcohol (PVA) is a low-cost synthetic polymer known as a promising cutaneous dressing [1,2]. However, its high moisture-wicking properties direct this material toward undesired microbiota growth. Several studies showed that the association with silver nanoparticles (AgNP) can enhance the polymeric matrix to inhibit the microbiota rise [1,3]. As pointed by Nuttelman and colleagues (2001) [4], PVA itself or containing AgNP cannot be applied as a skin substitute device since it does not possess cell or tissue affinity to induce healing or growth, due to its strong hydrophilic properties, creating an environment not favorable to cell adhesion. Tracking this thought, recent works showed the association of PVA and other natural polymers, such as collagen and hyaluronic acid, were able to gain a mimetic approach for skin healing [5,6,7]. However, one limiting challenge is the design of a complex dual-function device. To serve both as a dressing and as a matrix supply is the desire for viable low-cost industrial production. 

Developing countries, such as Brazil, have a healthy public system but with limited resources, are unable to offer high-end biotechnological devices to low-income patients.

Collagen possesses a high availability from assorted animal origins. The one obtained from bovine skin presents a low cost as compared to other sources. Its use as a natural biomaterial to favor wound healing is widely described in the literature [8], but on its own, the collagen itself lacks the ability to prevent infections or water loss from the injured skin. Rashid and colleagues (2019) [9], in the field of tissue engineering, reported that hydrogels from collagen or gelatin demand a chemical modification which could be achieved by covalent modifications of proteins using cross-linkable functional groups and chemical grafting of synthetic polymers onto protein backbones.

During the healing process, not only biocompatibility and infection prevention is important but the modulatory properties that regulate the intrinsic inflammatory responses and fibrosis formation must be carefully considered to avoid a chronic outcome [10]. Thus, evaluations with mesenchymal stem cells under in vitro and in vitro models associated with skin substitute devices represent a robust trial that evaluates a complete approach from anti-microbiological to its regenerative properties. Herein, a skin substitute mounted as a bilaminar device containing PVA-AgNP associated with reticulated collagen–hyaluronic acid (col-HA) membrane designed to induce skin regeneration, modulate inflammation, and minimize fungal and bacterial proliferation is presented. The newly regenerated tissue revealed that all skin layers were recovered after early periods of tissue repair.

## 2. Results

**Figure 1:**
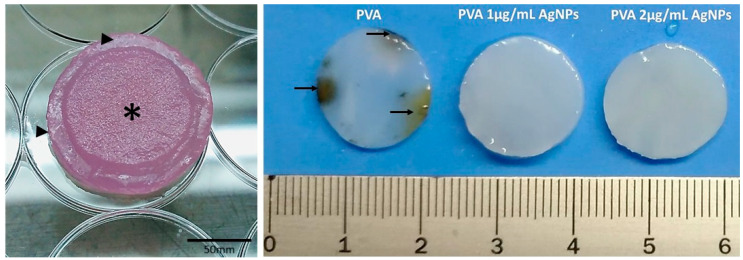
Left image shows macroscopic observations of the bilaminar device of PVA-AgNP (arrowheads) associated with the col-HA membrane (asterisk). The pink presentation is due to the culture medium absorption prior to the in vitro assays. Right image indicates the presence of PVA containing or not AgNP. After a few days under normal atmosphere, the free-AgNP samples presented a distinct fungal growth (arrows).

PVA is known for its unique high hydrophilic properties, and to avoid typical early fungal/bacterial growth after 1–3 days in normal environmental conditions, AgNPs were added into the device. The PVA (containing or not AgNP) films reached maximum swelling after 7 h of immersion, maintaining the same maximum swelling plateau around 163% up to 110 h. The AgNPs were tested at two different concentrations due to previous preliminary results (data not shown), which revealed that concentrations above 2 µg/mL of AgNPs started to present cytotoxicity. Thus concentrations of 1 and 2 were chosen for detailed microbiological evaluations. Figure 1 shows macroscopic observations of the unspecified fungi growth when PVA samples were not added to 1 or 2 μg/mL AgNPs. For the in vitro analysis, when PVA and the col-HA device were overlaid, generating a bilaminar device, one could observe with the naked eye the slight roughness in col-HA surface. No sealing factor was added to this bilaminar device. For the in vivo analysis, it was expected that the inner device would be integrated into healing tissue, while the PVA material would slowly dehydrate and naturally fall out under a normal atmosphere.

**Figure 2:**
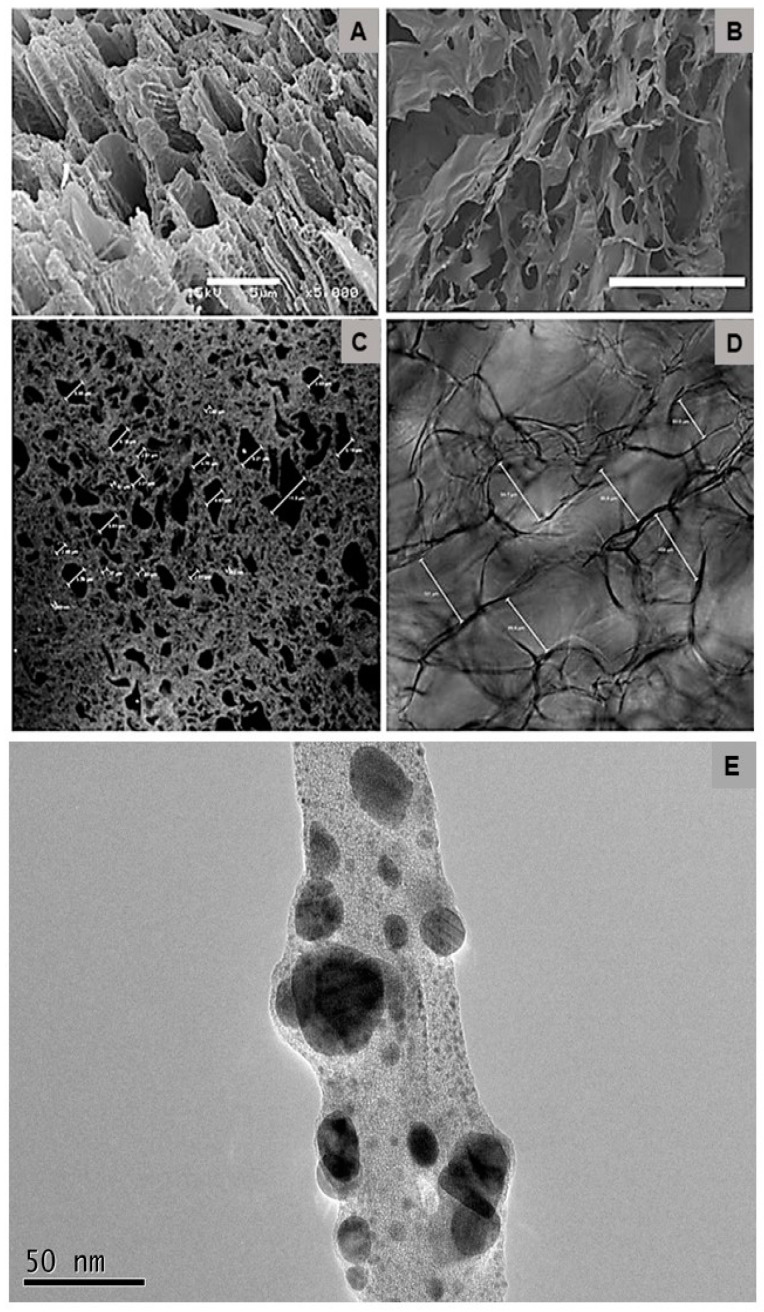
PVA (left) and col-HA (right) observed by SEM (**A**,**C**) and LSCM (**B**,**D**). HR-TEM observation of AgNP dispersed in Formvar coated Cu-grids (**E**). SEM bars represent 5 (**A**) and 100 μm (**B**). The assorted bars in LSCM depict the measurements made in samples, which varied from an average of 1 to 11 μm in PVA and 60 to 100 μm in col-HA.

The porous pattern is an important characteristic of a biomaterial designed as a skin substitute since tissue integration is enhanced. Thus, Figure 2 shows the surface properties of PVA-AgNP and col-HA by three different microscopy techniques. Since the porosity can be affected due to chemical sample processing for electron microscopy, its observations under any processing step were performed by LSCM. The porosity dimensions observed by LSCM (Figure 2B,D) present more accurate measurements as compared to SEM (Figure 2A,C). Additionally, HR-TEM observations revealed that the AgNPs presented dimensions that ranged on average within 5 to 20 nm (Figure 2E).

**Figure 3:**
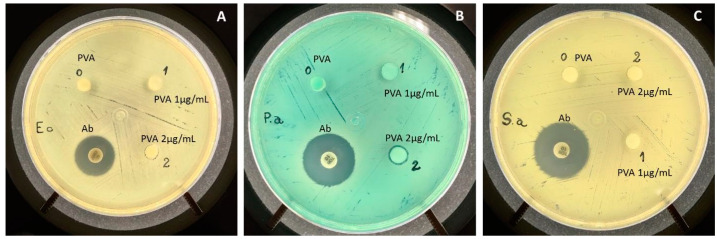
The antimicrobial activity of the PVA containing or not two concentrations of AgNP (1 and 2 μg/mL) was evaluated in the different bacterial species: (**A**) *Escherichia coli*; (**B**) *Pseudomonas aeruginosa*, and (**C**) *Staphylococcus aureus*. The Ab halo formation is represented by the standard antibiotic positive control.

Bacterial growth was not strongly inhibited in PVA containing AgNP, as can be observed in Figure 3. One can note a discrete halo indicating inhibition of *P. aeruginosa* at the concentration of 2 μg/mL of AgNP.

**Figure 4:**
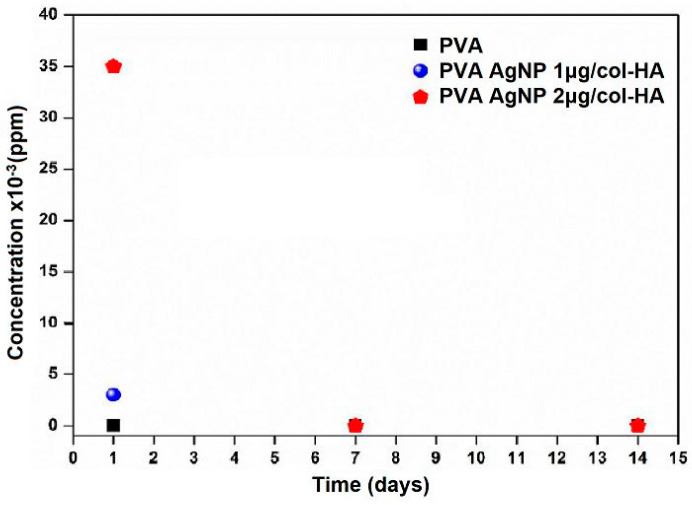
The AgNP release from PVA after 1, 7, and 14 days in DMEM culture medium detected by ICP-OES. This experiment was performed in duplicate.

The release of AgNP in PVA samples showed that after 1 day, rapid delivery of particles was detected at the highest concentration (2 µg/mL), while after 7 and 14 days (Figure 4), the release rate was near zero in all samples containing AgNP as compared to controls of pure PVA AgNP-free.

**Figure 5:**
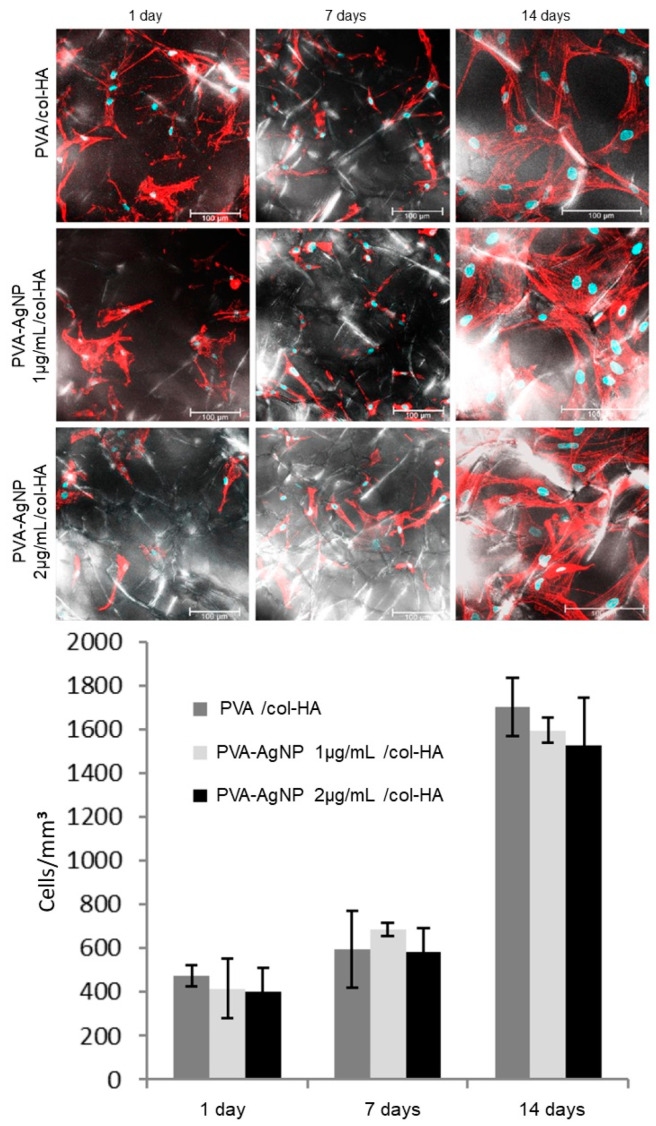
LSCM images of hMSCs adhered to PVA/col-HA containing or not AgNP at two concentrations. Images are shown as merged optical planes from the surface to a 100 µm depth reconstructed area. Bright/Dark background identifies the collagen distributed fibers shown by the confocal PMT-TRANS mode. The assorted polygonal-shaped cell morphology follows the random collagen distribution. Cell nuclei are identified by DAPI (blue) and actin cytoskeleton by Alexa Fluor 488 (red). The graphic shows the total cell count per area. Values are expressed as mean and standard deviation of a quintuplicate. No statistical significance was identified between groups of the same period. Bars 100 µm.

The in vitro evaluation of hMSC growth in samples containing AgNP at 1 and 2 µg/mL did not show any significant effect on cell growth quantification and on its typical morphology (Figure 5).

**Figure 6:**
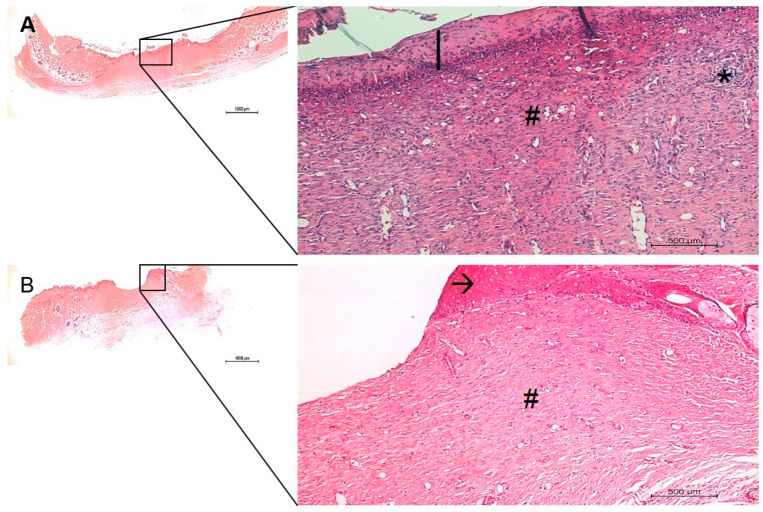
Histological images of the skin wound healing in negative controls, after 14 (**A**) and 28 (**B**) days. The right images are higher magnification areas from the entire lesion site depicted at left. (*) granulation tissue; (|) fibrin-leucocyte crust; (→) discontinued hyperplasic early epidermis; A dermis fibrous hyperplasia and increased telangiectasia are identified in (**A**, #), while lower hyperplasia and decreased telangiectasia are evident in (**B**, #). Bars 1000 µm.

**Figure 7:**
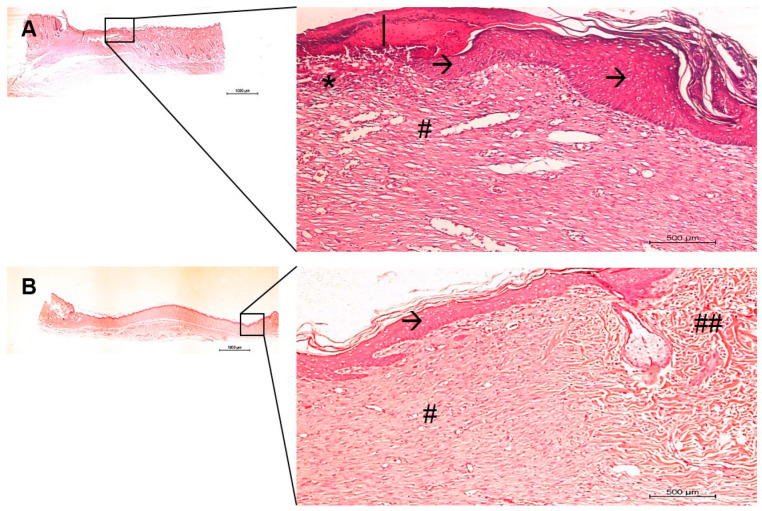
Histological images of the skin wound healing implanted with PVA-AgNP, after 14 (**A**) and 28 (**B**) days. The right images are higher magnification areas from the entire lesion site depicted at left. (*) granulation tissue; (|) fibrin-leucocyte crust; (**A**, →) discontinued hyperplasic early epidermis; (**B**, →) Continuous new epidermis; A dermis fibrous hyperplasia and increased telangiectasia are identified in (**A**, #), while lower hyperplasia and decreased telangiectasia are evident in (**B**, #), additionally, older areas bordering the wound limits shows normal loose tissue (##). Bars 1000 µm.

**Figure 8:**
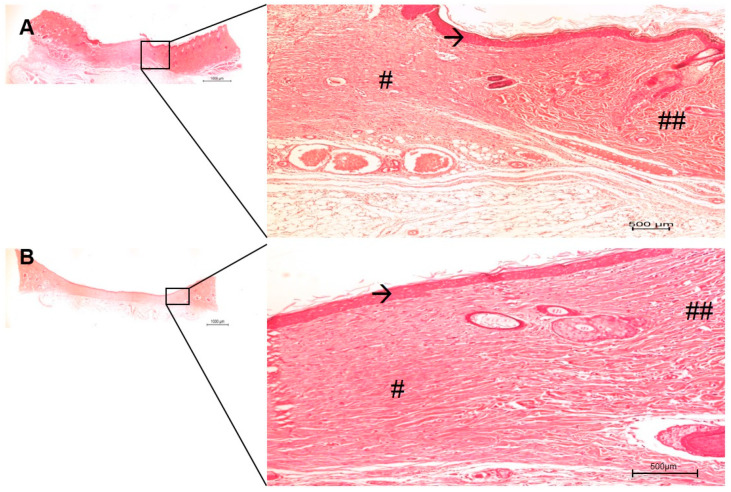
Histological images of the skin wound healing implanted with PVA-AgNP/col-HA, after 14 (**A**) and 28 (**B**) days. The right images are higher magnification areas from the entire lesion site depicted at left. (→) early epidermis; the new dermis (#) presented a fibrous pattern quite similar to the old dermis (##). Bars 1000 µm.

**Figure 9:**
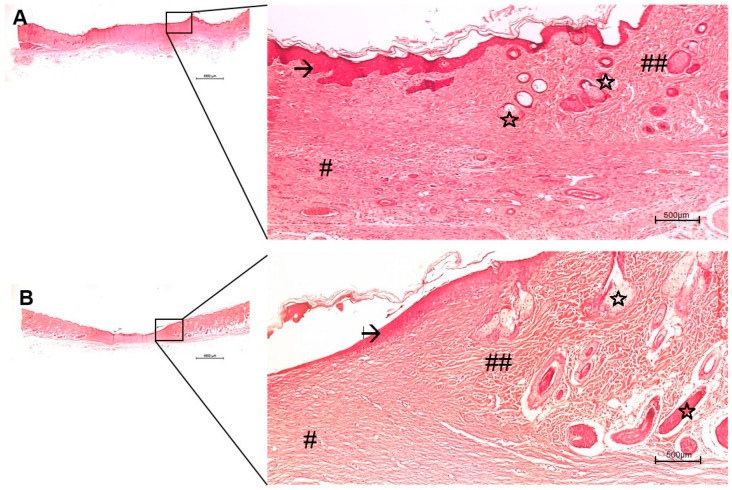
Histological images of the skin wound healing implanted with PVA-AgNP/col-HA/hMSC, after 14 (**A**) and 28 (**B**) days. The right images are higher magnification areas from the entire lesion site depicted at left. (→) early epidermis (with crests in A); the new dermis (#) is less than the fibrous pattern (##) where pilous follicle structures were more richly identified (stars). Bars 1000 µm.

The in vivo histological observations showed a gradual tissue recovery progress of the wound healing the more complex the device design was (from pure PVA to PVA containing AgNP + col-HA + hMSCs; Figure 6, Figure 7, Figure 8 and Figure 9, respectively). Based on the results obtained in vitro, the AgNP concentration chosen to be evaluated in vivo was 2 µg/mL. The two panels of observations (panoramic view of the entire lesion) and the inserted areas (detailed characteristics of cells and tissue healing) revealed that in all groups, only negative controls presented strong granulation tissue and large fibrin-leucocyte crusts. A discrete crust and inflammatory cells were observed in PVA-AgNP group 14 days after injury, while no inflammatory sign was identified in all samples containing col-HA. The structures associated with pilous follicles were remarkably noted in lesion borders when hMSCs were present, although the one containing col-HA presented the highest epidermis regeneration, which presented 100% of epithelial cells coating in the wound surface.

**Figure 10:**
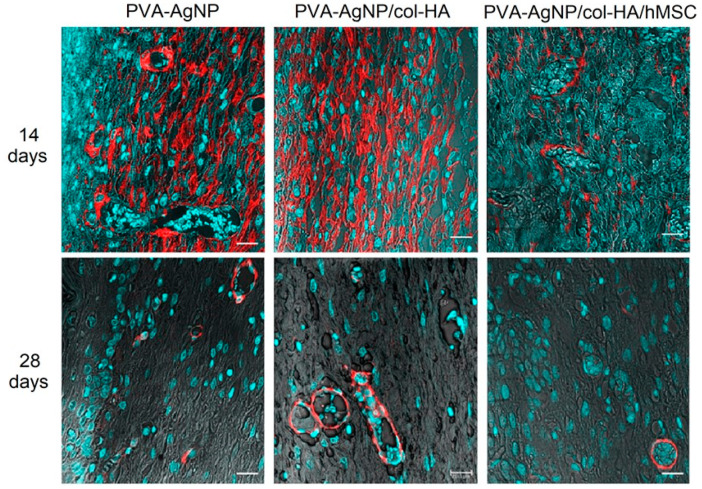
LSCM histology sections of the skin wound healing immunolabeled for α-SMA (red). Both myofibroblasts and microcirculation vessels can be distinctly identified. The cell nuclei are identified by DAPI (blue). Loose stromal tissue is observed by the PMT-TRANS mode (grayscale contrast structures). Bars 10 μm.

**Figure 11:**
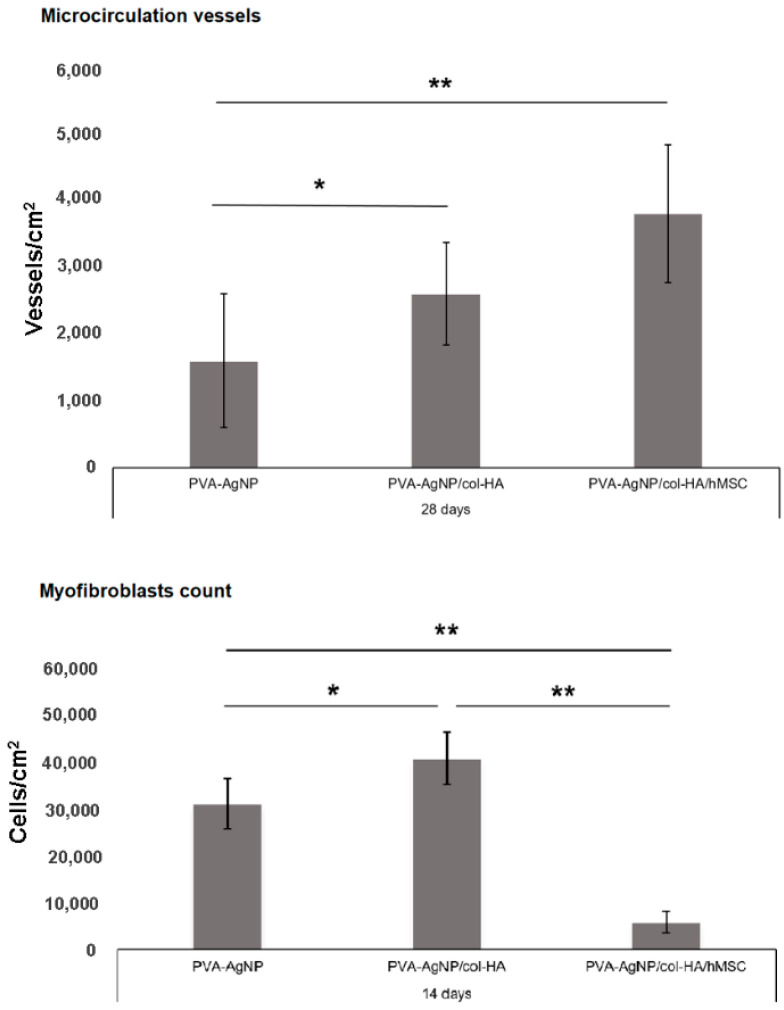
The quantification of microcirculation vessels and myofibroblasts were obtained from LSCM images labeled by α-SMA. The values are expressed as means and standard deviation of two samples for each group in an area of 1 cm^2^ observed. Quite or very significant changes within samples are identified as (*) and (**), respectively.

The myofibroblasts are cells typically differentiated from fibroblasts and are recruited to decrease the skin wound size. Due to their contractile properties, myofibroblasts are known to be responsible for the fibrous macroscopic presentation of the scars. Although cells are important to give an early response to close the injured area minimizing infections, it is important to regulate their activity properly to avoid excess fibrosis. In this way, the myofibroblasts were identified in all samples only after 14 days of injury, but strikingly fewer in the samples implanted containing hMSCs (Figure 10). Since the α-SMA also detects blood vessels due to its wall containing smooth muscle cells and pericytes (also labeled by the α-SMA), the presence of the microcirculation was identified and evaluated. The images were processed to quantify myofibroblasts and vessels by the LAS X software measuring/detection module (Figure 11).

## 3. Discussion

Patients with burned skin can be exposed to the invasion by microorganisms that can lead to death from septicemia [11]. AgNP is known for its antimicrobial properties [12]. As reported by Lu and colleagues (2013) [13] and Dong and colleagues (2019) [14], the more nanometric the particle is, the more effective it is in inhibiting the growth of microorganisms. Thus, the particle size of the AgNP here obtained ranged from an average 5 to 20 nm (Figure 2E). When added to the PVA, it presented strong fungal inhibitory growth (Figure 1) with a mild bacterial inhibition, as shown by the microbiological assay by disk diffusion that demonstrated the presence of a small halo only in the PVA-AgNP 2 μg/mL sample (Figure 3). Mahmoudi and Serpooshan (2012) [15] reported that during AgNP oxidation, the Ag^+^ can interact with molecules of the bacterial membrane promoting its depolarization leading to its death. In addition, it was demonstrated that the inhibitory mechanism of silver ions in microorganisms promotes the loss of DNA replication and gene inactivation [16].

The ICP-OES analysis revealed that during the first 24 h, a rapid release of Ag^+^ was delivered to the cell culture medium (Figure 4). As reported in studies [17,18,19], Ag^+^ can present in vitro cytotoxicity, but the in vitro results showed that both concentrations tested (1 and 2 µg/mL in PVA) did not affect cell growth and its phenotypic features (Figure 5). PVA itself is highly hydrophilic, which presents a dual property that can be both positive and negative as a curative to wound healings. In this way, the high hydration properties avoid water loss and dehydration but support a good environment for microorganism growth that could produce infections [20]. Therefore, PVA can usually be associated with other natural polymers, such as chitosan and other antimicrobial drugs, in order to increase its biocompatibility as a dressing, minimizing bacterial growth [21]. The results of early Ag^+^ delivery in medium and high hMSCs growth in vitro after long-term culture (14 days), were important parameters that guided us to use AgNP at the concentration of 2 µg/mL in the in vivo assays.

The morphological analysis showed that the presence of AgNP in the PVA membrane did not interfere with the porosity of the device (Figure 2). The presence of pores in the membrane is important because it allows an increase in the oxygen concentration in the lesion, which is necessary for several cellular processes, such as phagocytosis, mitosis, and expression of growth factors, that are essential for injury repair [22]. The col-HA scaffold was also extremely porous, with interconnected porosity. Such porous structures provide interaction between cells, and favor the passage of cytokines and growth factors [23]. Scaffold pore diameters in the range of 60 to 100 μm are considered an optimum size for cellular migration and angiogenesis, as reported elsewhere [24]. The in vitro LSCM observation showed that hMSCs adhered following the orientation of the col-HA fibers. Interestingly, some first preliminary results of cells cultured only in pure PVA or PVA-AgNP revealed a zero rate of hMSCs adhesion (data not shown). As already reported by Marin and colleagues (2014) [25], PVA derivatives can be applied to assorted clinical purposes, but there is a lack of information on its direct effectiveness in cell adhesion in the pure form. Probably due to its high hydrophilic condition, it prevents the formation of focal contacts by cells leading to nonattachment.

The in vivo approaches showed that the PVA-AgNP/col-HA/hMSC and PVA-AgNP/col-HA groups presented the most accurate results as compared to PVA-AgNP and negative control, due to the presence of accelerated cicatricial remodeling in the dermis and hypodermis without fibrosis, resulting in a similar tissue of the original hypodermis. The mild regeneration identified in the PVA-AgNP group as compared to negative controls, indicate that the high swelling property of PVA (163%), apart from the physical protection to avoid microorganism growth, can also retain the wound exudate to avoid dehydration. The presence of collagen containing HA was the main characteristic that determined increased healing since the collagen porosity facilitates nutrient transportation, migration to and endothelial cell infiltration into the wound local, favoring angiogenesis [24,26]. The HA is formed by D-glucuronic acid and N-acetyl-glucosamine and, when degraded, it delivers these two essential substances for the tissue regeneration process [27]. West and colleagues (1985) [28] demonstrated that the degradation products of HA are pro-angiogenic factors. Furthermore, the HA is a preferential structure of fibroblast adhesion during wound healing. Its specific property of attracting water and stimulatory factors favors the diffusion of the molecules through to the injured site [29]. The effectiveness in favor of skin healing for PVA-AgNP/hMSC and PVA-AgNP/col-HA can be depicted as the complete coverage of epidermis in the lesion site in the early period of 14 days after injury. The group with hMSC presented a cicatricial remodeling more pronounced than the group without cells.

Przekora (2020) [30] reviewed artificial skin grafts and found they favored the formation of appendages of the integument with controlled antibacterial activity, which is a recent challenging trend in the engineering of biomaterials. Such characteristics can be enhanced by the presence of hMSCs. These cells have an active role in the inflammation, proliferation, and cicatricial remodeling phases [31]. The hMSCs coordinate the activity of inflammatory cells, inhibit the secretion of pro-inflammatory cytokines, and stimulate the secretion of anti-inflammatory cytokines in the inflammatory process [32,33]. In relation to the migration, proliferation, and remodeling phases, the hMSCs stimulate angiogenesis and induce the migration of epidermal cells and fibroblasts into the wound through paracrine signaling. In addition, the hMSCs regulate the deposit and organization of extracellular matrix made by fibroblasts, which accelerates the proliferation process, cicatricial remodeling, and angiogenesis [31,34]. However, the use of allogeneic or autologous cells in engineered biomaterials is controversial due to its availability and limitations [35]. Thus, adipose-derived MSCs are an abundant source under low invasive surgical techniques that could be used in cases where large areas of skin repair would be necessary. The tissue dissociation into free expanded MSC cells is a simple technique that can be achieved in a few days, and after its association with bioengineered devices, can be reimplanted in the desired area.

Myofibroblasts cell type can be characterized by the α-SMA identification in the cytoskeleton, and this labeling can also be a marker to identify microcirculation due to the presence of smooth muscle cells and pericytes in vessels, although it will only label the microcirculation vessels as arterioles and venules, but does not label capillaries [36]. However, the labeling of arterioles and venules was considered enough as identification of microcirculation vessels and can promptly represent a sign of healing, since arterioles and venules are usually identified in late phases of the healed dermis, while in this work, such vessels were easily identified in the early phases (14 days) after injury. As reported by Frank, Madlener, and Werner (1996), the low expression of TGFβ-3 in the healing process can be attributed to myofibroblasts that play a role in fibrosis formation [37]. Interestingly, low myofibroblasts’ presence and increased angiogenesis identified in the early stages (14 days in Figure 10 and cell count in Figure 11) indicate clearly that the hMSCs play a role in reducing fibrosis and favor the microcirculation formation, as already proposed in a previous study [38]. Such conditions can be directly related to low scar fibrotic formation, while higher angiogenesis can achieve improved support of growth factors accelerating tissue healing.

The third-degree model of skin burn induced de-commitment of skin appendages. Yet, the presence of abundant hair follicles, including sebaceous glands here detected (Figure 8 and Figure 9), is an indicator that the bilaminar device fulfills the desired properties as a dual function, such as wound dressing, infection protection, and as an integrative source of skin-derived matrix to accelerate the functionalities of the tissue. The bilaminar device here presented was designed to be overlaid in two sheets, free of any binding properties between the two layers. After being implanted, the inner col-HA layer containing cells was rapidly integrated into the healing tissue, while the PVA-AgNP formed a physical barrier to avoid infections, in addition to the hydration activity. The results obtained by ICP-OES (Figure 4) revealed that AgNP was delivered within 24 h. This fact could limit the effectiveness of the time needed to keep the outer layer of PVA-AgNP covering the wound due to the fast decrease in the antibacterial activity of the device. In this matter, in a clinical trial, the PVA-AgNP layer should be exchanged as soon as the inner layer is integrated into the tissue. However, other limiting factors in clinical use, such as the availability of an autologous stem cell source, injury extent, pre-existing topical infection, and vascular commitment due to necrosis, still demand caution and suggest only considering a skin substitute useless previous evaluation of the wound characteristics has been conducted. Nevertheless, more details around a clinical trial are required, but forward studies of this device are very promising.

## 4. Materials and Methods

### 4.1. Synthesis of PVA-AgNP/col-HA

A solution of 8% *w/v* of PVA (Sigma-Aldrich^®^, St. Louis, MO, USA) in distilled water heated to 80 °C was placed under stirring. After that, the PVA was added to 0, 1, or 2 μg/mL of a AgNP solution at room temperature. Prior to addition to PVA, the AgNP was freshly obtained according to Higa and colleagues (2016) [39] and did not use any toxic solvent, which generates minimal residues. The AgNP suspension was prepared chemically via the reduction in silver nitrate with sodium citrate, as detailed in [39]. The PVA-AgNP hydrogel was placed on plates submitted to −18 °C temperature for 16 h, followed by cycles of cooling/thawing for 8 h at room temperature. These cycles were repeated twice. Swelling tests were performed, and some samples were cut into 20 (l) × 10 (w) × 0.3 (t) mm^3^ sections and immersed under a pH 7.4 saline solution for up to 110 h at 37 °C. Samples were dried to remove the surface moisture after removal from the saline and weighed by a high precision analytical balance to evaluate the mass change during the swelling. The percentage of weight variation absorbed by each specimen was calculated from its initial weight (*w*_0_) and its weight after absorption (*w_t_*), as follows in Equation (1):

(1) $$swelling\left(\%\right)=100\cdot \left(\frac{W_{t}-W_{0}}{W_{0}}\right)$$

The collagen solution from bovine skin (code C2124, Sigma-Aldrich^®^, St Louis, MO, USA) and hyaluronic acid (HA) (code 9067-32-7, Chengzi Life Science Co, Ltd., Beijing, China) were used in this work. The HA powder was dissolved in dH_2_O at 6 mg/mL. The Col-AH solution was obtained through a dissolution of Col and HA solution at the ratio of 9:1, respectively. The solution of col-HA was deposited into 10 cm diameter Petri dishes, frozen at −20 °C for 24 h, and lyophilized for the same period. Afterward, it was submitted to a reticulation process under immersion on 95% ethanol solution supplemented with 50 mM 1-ethyl-3- (3-dimethylaminopropyl) carbodiimide (EDC) under neutral pH, for 7 h at RT. Each device was washed twice for 1 h with 0.1 M disodium phosphate, twice for 1 h with 1 M sodium chloride, 6 times for 24 h with 2 M sodium chloride, and 10 times under intense magnetic stirring agitation with bidistilled water (2 L for each bath) to remove all EDC residues. The obtained col-HA membranes were dried at RT for 24 h under UV-C radiation. After being dried, the PVA/AgNP was 0.5 mm thick, while the Col-AH had 0.2 mm.

### 4.2. Surface and Morphology Characterization of AgNPs/PVA-AgNP

Due to the size of the AgNP interfering with microbiological activity, as already reported in the study by [40], the AgNP diameter was characterized by Transmission Electron Microscopy (TEM). In this regard, a drop of the solution containing AgNP was placed on a copper metal grid containing Formvar films and examined in TEM JEOL 2100F 200 kV.

Since the PVA-AgNP is strongly hydrated, the material was dehydrated with an ethanol growing series and critical point dried. The collagen-based scaffold was not submitted to the dehydration process. Both materials were submitted to a gold sputter coater and analyzed to Scanning Electron Microscopy (SEM) (JEOL JXA-840A).

Due to possible interferences attributed to dehydration in electron microscopy analysis, the PVA-AgNP was observed in its natural state just after obtaining it without any chemical processing in a laser scanning confocal microscope (Leica TCS SP8) under PMT-TRANS mode at 430–490 nm of signal detection. The LAS AF software was used to obtain the images that were pseudo-colored in blue and to digitally measure the pore widths.

### 4.3. Isolation and Cell Culture

The human mesenchymal stem cells (hMSC) (also commonly named adipose-derived stem cells, ADSC) were isolated from the discarded adipose tissue obtained from liposuction of a female patient submitted to aesthetic plastic surgery (approved by the Unicamp Research Ethics Committee no. 1,650,961). The aspirated samples were collected in 50 mL syringes and immediately sent to the isolation procedures. The tissue was digested with trypsin solution diluted in DMEM (not serum-supplemented) at the ratio 1:1 (used 20 mL for each solution in 50 mL falcon tubes) for 30 min under rotative shaking in a cell culture incubator. After this process, the sample was added to 5 mL of FBS and resuspended, centrifuged under 4 °C for 15 min at 1500 rpm. All supernatant was carefully removed (dense clots of fat and remaining blood), and the pellet was resuspended in DMEM containing 10% of fetal bovine serum and custom antibiotics. The cells were seeded in 75 cm^2^ cell bottles and maintained under a cell culture incubator with 5% CO_2_ atmosphere at 37 °C. The medium was changed every day for 7 days to remove the remaining blood cells. The hMSCs have the ability to differentiate to adipocytes and osteoblasts, due to this, the cells were characterized after isolation with the inductors medium StemPro™ Adipogenesis Differentiation Kit (ThermoFisher, Massachusetts, USA, A1007001) and StemPro™ Osteogenesis Differentiation Kit (ThermoFisher, Massachusetts, USA, A1007201) for 14 days. The analysis was achieved using the Oil Red dye, which evidences the cumulative intracellular lipids and, Alizarin Red that identifies the calcium deposits under osteogenesis (the data is not shown since the results obtained are a standard method to characterize the isolated cells as hMSC lineage).

### 4.4. Analysis of Ag^+^ Liberation in the Culture Cell

The quantification of Ag^+^ was analyzed after 1, 7, and 14 days by PVA-AgNP membrane delivery. The supernatant from the hMSC cultivation in the bilaminar device was used for this quantification. The samples, PVA only, PVA-AgNP 1 μg/col-HA, and PVA-AgNP 2 μg/col-HA, were digested with nitric acid and quantified by ICP-OES (Optima 8000 ICP-OES spectrometer Perkin Elmer, Massachusetts, USA).

### 4.5. Cell Behavior in PVA/col-HA with AgNPs

The material (PVA/col-HA; PVA containing AgNP at 1 or 2 μg/mL/col-HA) was sterilized with 70% ethanol for 1 h, washed with phosphate buffer saline (PBS) adapted for 4 h in DMEM at 37 °C. The cells were seeded in a material at the concentrations of 3 × 10^4^ cells/sample. After 1 and 7 days under culture, their proliferative pattern phenotype was evaluated both in the material with PVA-AgNP/col-HA and with hMSCs. It is known that the cells do not adhere to PVA [41,42], thus the cell seeding was performed at the side of the device containing the col-HA. After each culture period, the cells were fixed with paraformaldehyde (PFA 4%) and the nuclei were labeled with 4′,6-Diamidine-2′-phenylindole dihydrochloride (DAPI) (Sigma-Aldrich™, St. Louis, MO, USA) and analyzed by Laser Scanning Confocal Microscopy (LSCM) (TCS SP8 model, Leica MicroSystems™, Wetzlar, Germany). According to the images obtained, the cells’ nuclei were counted using the ImageJ software. Regarding the morphology analysis, the cells were fixed with PFA 4% and permeabilized with Triton-X 1%. Therefore, the cytoskeletons were labeled with phalloidin conjugated with Alexa Fluor 647 (Sigma-Aldrich™, St. Louis, MO, USA). Images were analyzed by LSCM under PMT mode, at 53 μm of pinhole aperture, using laser line 638 at the emission detection range of 650 to 750 nm for Alexa Fluor detection, and laser 405 at the emission range of 420 to 460 for DAPI detection.

### 4.6. Microbiological Assay

Pure PVA membranes containing different AgNP concentrations were aseptically cleaned with 70% ethanol for 30 min. Afterward, the membranes were washed with sterile PBS, and their antimicrobial potential was analyzed by the disk diffusion technique for 24 h (Kirby Bauer method), using *Staphylococcus aureus* (ATCC25923), *Pseudomonas aeruginosa* (ATCC27853), and *Escherichia coli* (ATCC25922). The bacteria were incubated in Müeller–Hinton agar at 37 °C for 24 h. Tobramycin disks were used as positive control. Additionally, unspecified fungal growth was evaluated by leaving samples under normal atmospheric air conditions for up to 7 days.

### 4.7. In Vivo Study

**Figure 12:**
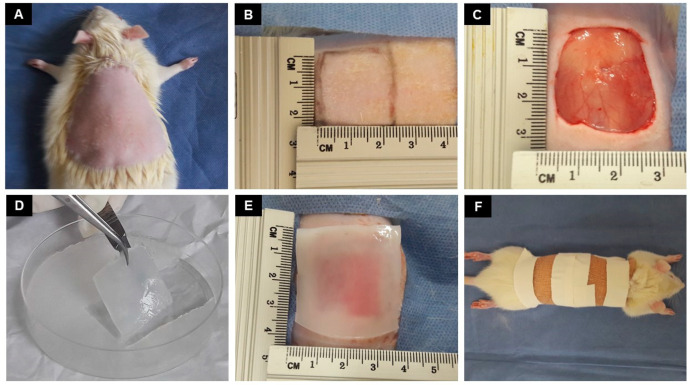
Images of the surgical procedure: (**A**) rat dorsal trichotomy; (**B**) definition of injury area; (**C**) dissected skin area; (**D**) sample membrane (PVA-AgNP in this image); (**E**) implanted area with the membrane; and (**F**) the dressing placed over the implant.

All 30 Wistar rats used, both genders, 3 months aged, 250–300 g weight, were divided into 4 main groups according to the period of implant analysis (14 and 28 days), each period was divided into 4 groups: negative control (lesion only), PVA-AgNP, PVA-AgNP/col-HA, and PVA-AgNP/col-HA/hMSCs. The group that contained cells was seeded with 1 × 10^5^ cells/cm^2^ and cultured for 7 days before implantation. The rats were acquired by ICB/USP and maintained at the laboratory animal household of FCMS/PUCSP under approval by the Ethics Committee on Animal Use of the Faculty of Medical and Health Sciences of the PUCSP, number 2016/56). For the surgical procedure (Figure 12), the animals were weighed and submitted to anesthesia with Ketamine hydrochloride 10% (40 mg/kg) and Xylazine hydrochloride 2% (5 mg/kg) solution per body weight [43]. After trichotomy of the dorsal region near the neck (Figure 12A), followed by an aseptic bath with iodine-povidone, each animal was dissected (2 × 2 cm^2^), removing all skin layers but leaving the muscle fascia intact (Figure 12B,C). The lesion obtained simulated a third-degree burn. Minutes after this procedure, the wounds were implanted to membranes of PVA-AgNP, PVA-AgNP/col-HA, or PVA-AgNP/col-HA/hMSCs (Figure 12D,E), except the negative control, that was not exposed to any curative bandage. After implantation, the dressing was placed over the implant to prevent infections (Figure 12F).

The rats were kept under careful observation every 12 h for the first 7 days after implantation. Elizabethan collars were used for the first 48 h to avoid removing the implanted samples. All curatives fell off after 2 or 5 days after their addition. Any other curative that might be lost before a minimum of 48 h period led to removing the animal from its group. After 14 and 28 days from implantation, the rats were sacrificed by halothane inhalation, and the implanted healing tissue area (treatment groups and negative control group) was fixed in formalin 10% for a period of 24 h under 4 °C.

### 4.8. Histology and Immunohistochemistry

The samples were prepared for histological analysis according to the light microscopy standard techniques, using paraffin as a means of inclusion. Samples were submitted to an increasing series of ethanol dehydration for 30 min each, followed by diaphanization with xylene in a two-step series of 30 min each. The materials were immersed in two baths of paraffin (Histosec-Merck^®^, Darmstadt, Germany) for 30 min and included as performed afterward. The histology blocks were cooled and microtomized (Leica^®^ RM2245, Wetzlar, Germany) in 3–5 µm thickness. The slides were stained with hematoxylin–eosin (HE) for light microscopy observations or processed for immunohistochemistry to laser scanning confocal microscopy (LSCM) analysis. Tissue sections were primarily labeled with monoclonal Anti-α-Smooth Muscle Actin (α-SMA) antibody produced in mouse (Sigma-Aldrich #A2547, St. Louis, MO, USA) and secondary labeled with Goat Anti-Mouse conjugated with Alexa Fluor^®^ 647 (Abcam #ab150115, Cambridge, UK). Slides were mounted with a Fluoroshield with DAPI to avoid image background effects and to identify cell nuclei, respectively. The healing tissue area observed by LSCM was the early dermis adjacent to the new epidermis. The α-SMA identification at the immunohistochemistry sections was used to quantify myofibroblasts and microcirculation vessels. The definition of the level of hyperplasia/telangiectasia and other features was defined according to visual comparison with negative controls. The results were expressed as mean with standard deviation and analyzed by the ANOVA analysis of variance test by the Tukey test; *p*-values < 0.05 indicated statistical difference.

## 5. Conclusions

The developed bilaminar device, composed of PVA-AgNP/col-HA with different concentrations of AgNPs, was able to grow the hMSC without interference on the adhesion, proliferation, and morphology. Pure PVA itself does not possess antimicrobial activity, but when added to AgNP, it favors a rapid Ag^+^ release after 1 day, which inhibits the microbiota growth in vitro. When added to col-HA and hMSC, this device strongly fastens wound healing in the in vivo model evaluated. Additionally, the hMSCs interfere in the myofibroblasts’ formation and also enhance microcirculation. The PVA-AgNP containing or not the col-HA and hMSC presented the necessary characteristics to be applied as a substitute skin since no infection signals were detected in the injury. Further, it prevented water loss and improved the full growth of a neo epidermis and dermis with reduced fibrosis formation.

## Data Availability

Partial data of this work is available in a publicly accessible repository that does not issue DOIs. Publicly available datasets were analyzed in this study. This data can be found here: http://repositorio.unicamp.br/jspui/bitstream/REPOSIP/333244/1/Domingues_JulianaAlmeida_D.pdf.
